# Divergent Trajectories of PSA and Testosterone During Sexual Maturation in Male Dromedary Camels (*Camelus dromedarius*): Implications for Androgen-Independent Prostate Biomarker Dynamics

**DOI:** 10.3390/ani16111740

**Published:** 2026-06-05

**Authors:** Hafiza Aidoudi, Soundes Akriche, Maria Chikha, Djallal Eddine Rahmoun, Carlos Iglesias Pastrana

**Affiliations:** 1 Laboratory of Sciences and Technics of the Livings, Institute of Agricultural Sciences and Veterinary Sciences, Mohamed Cherif Messaadia University—Souk Ahras, Souk-Ahras 41009, Algeria; hafizaaidoudi@gmail.com; 2 Pathological Physiology Department, Saint-Petersburg State University of Veterinary Medicine, 196084 Saint Petersburg, Russia; soundes.akriche@yandex.ru; 3 Department of Veterinary Science, Faculty of Natural and Life Sciences, University Echahid Hamma Lakhdar, El Oued 39000, Algeria; mariachikha1997@gmail.com; 4 Institute of Agriculture and Veterinary Sciences, University of Batna, Batna 05010, Algeria; deddine44@gmail.com; 5 The Andalusian Institute for Agricultural, Fishing, Food and Ecological Production Research and Training (IFAPA), Alameda del Obispo Center, 14004 Córdoba, Spain

**Keywords:** biomarker, dromedary camel, prostate gland, prostate-specific antigen, sexual maturation, testosterone

## Abstract

The relationship between prostate-specific antigen (PSA) and testosterone during sexual maturation in male dromedary camels remains poorly understood, despite its clinical importance in prostate health monitoring and reproductive management. In 50 intact male dromedaries divided into five age groups, we looked at the longitudinal changes of these biomarkers. Testosterone showed a substantial correlation with age (r = 0.89), although PSA only showed a modest connection (r = 0.41). Phase-specific correlations between PSA and testosterone were non-significant, ranging from slightly negative (r = −0.39) during the transitional phase to modestly positive (r = 0.40) after puberty. In conclusion, these findings demonstrate a distinct divergence between PSA and testosterone trajectories during camel maturation, which may reflect developmental and functional changes associated with prostatic cellular differentiation and functional maturation during puberty, with the possibility of specific regulatory mechanisms that may not be entirely androgen-dependent. Establishing these age-specific profiles provides an essential biomarker framework to enhance reproductive health assessment and veterinary management strategies in dromedary camels.

## Simple Summary

Understanding how the prostate develops in male dromedary camels (*Camelus dromedarius*) is important for improving their reproductive management and health monitoring. However, little is known about how key biological markers related to the prostate change as camels grow. In this study, we examined two markers in 50 male camels of different ages: one produced by the prostate and another linked to male hormones. We found that these markers do not follow the same pattern during growth. While the hormone level increased steadily with age, the prostate marker showed an unexpected drop during a transitional stage before rising again in adulthood. This suggests that, unlike in humans, the prostate in camels may not be controlled only by male hormones. These findings provide useful reference values for different ages and improve our understanding of camel biology. This could help veterinarians and breeders better monitor animal health and manage breeding programs more effectively.

## 1. Introduction

The dromedary camel (*Camelus dromedarius*) occupies a pivotal role in arid and semi-arid ecosystems, serving as a critical resource for transportation, milk production, and meat in regions where few other livestock species thrive [1]. Despite its economic and cultural importance, the reproductive biology of male camels remains understudied compared to other domesticated species, particularly concerning endocrine regulation and prostate health biomarkers [2]. While prostate-specific antigen (PSA) has been extensively characterized in humans as an androgen-dependent marker of prostate function [3], its dynamics in camelids exhibit distinct species-specific patterns that challenge conventional mammalian models.

Male camel reproduction is governed by highly unique anatomical and physiological adaptations, including perineal testicular positioning, delayed puberty, and pronounced seasonal variations in sexual activity [4]. Unlike non-seasonal domesticated livestock, male camels experience a distinct seasonal estrous known as the ‘rutting season’ or ‘rut’, during which neuroendocrine shifts drive intense territorial behavior and mating proficiency [5]. Testosterone, the primary androgen driving sexual maturation, follows this well-documented seasonal rhythm in adult camels, with peak concentrations during the rutting season [6]. However, the developmental trajectory of testosterone from birth through sexual maturity, and its relationship with prostate biomarkers like PSA, has not been systematically investigated. This gap is particularly significant given the divergent reproductive strategies between camels and other ungulates, including prolonged prepubertal periods and delayed sexual maturity.

Furthermore, understanding species-specific differences in prostate biology necessitates looking beyond human models to traditional domestic livestock. In cattle and sheep, true structural counterparts to human PSA are absent; instead, their prostates secrete specific tissue kallikreins and unique esterases [7,8]. The few available reports on camelid PSA suggest markedly lower baseline concentrations compared to humans, but the regulatory mechanisms governing its expression remain speculative [9]. In humans, PSA production is tightly coupled with androgen receptor signaling, making it a sensitive indicator of both physiological and pathological prostate activity [3]. Whether this relationship holds true in camels—a species with fundamentally different reproductive anatomy, a unique seasonal breeding pattern, and divergent endocrine regulation—has not been empirically tested.

We hypothesized that PSA and testosterone would follow divergent developmental trajectories in male dromedary camels, reflecting species-specific uncoupling of androgen signaling and prostate biomarker dynamics. This hypothesis challenges the conventional paradigm of androgen-dependent PSA regulation observed in humans and most mammalian models. To test this, we conducted a study measuring serum PSA and testosterone concentrations across five critical developmental stages (<1 to 4–5 years), capturing the complete transition from neonatal immaturity to sexual maturity. Our primary objectives were to establish age-specific reference intervals for these biomarkers, characterize their temporal relationship during sexual maturation, and evaluate the clinical utility of PSA as a prostate health marker in camelids.

This study makes three key contributions to camelid reproductive medicine. First, it provides the first comprehensive dataset on PSA and testosterone dynamics during male camel development, filling a critical knowledge gap in species-specific endocrinology. Second, it identifies a previously unrecognized pattern of PSA fluctuation that inversely correlates with testosterone surges during puberty, suggesting androgen-independent regulatory mechanisms. Third, the established reference intervals enable evidence-based interpretation of these biomarkers in clinical and breeding contexts, with direct applications for infertility diagnosis and prostate health monitoring.

## 2. Materials and Methods

The study employed a cross-sectional design to evaluate the developmental trajectories of prostate-specific antigen (PSA) and testosterone in male dromedary camels. Technical details regarding animal selection, sample collection, laboratory assays, and statistical analysis are described in the following subsections.

### 2.1. Animal Selection, Housing and Ethics

Fifty clinically healthy intact male dromedary camels were enrolled from traditionally managed herds in southern Algeria. The study protocol followed national veterinary care standards, and formal institutional ethical approval was waived due to the completely non-invasive, field-based nature of the blood sampling, which was performed during routine veterinary checks with the explicit verbal and written consent of the animal owners. The camels were maintained under a semi-extensive Saharan management system, grazing on natural pastoral rangelands supplemented with wheat straw and commercial concentrate pellets during periods of low forage availability, with free access to water. Animals were stratified into five age groups: (<1) year (neonatal/prepubertal), (1–2) years (early prepubertal), (2–3) years (transitional/pubertal), (3–4) years (post-pubertal), and (4–5) years (mature). Inclusion criteria required normal physical examination findings, absence of reproductive tract abnormalities, and body condition scores (≥2.5) on a 5-point scale. Strict exclusion criteria were applied; animals were excluded if they showed clinical signs of acute or chronic systemic illness, musculoskeletal disorders, injuries, or any structural abnormalities of the reproductive tract detected during standard physical examination and external palpation of the testes and scrotum.

### 2.2. Blood Sampling and Processing

Blood samples were collected via jugular venipuncture between 07:00 and 10:00 h during the seasonal rutting (estrous) period of the camel to minimize potential diurnal variation in hormone concentrations and to ensure baseline physiological stability before daily environmental and thermal stress peaks [10,11]. Serum was separated by centrifugation at 3000× *g* for 15 min (Centrifuge 5810 R, Eppendorf SE, Humb ambient temperature) after 30 min of clotting at ambient temperature. Aliquots were stored at −80 °C (TSX Series −80 °C Ultra-Low Freezer, Thermo Fisher Scientific Inc., Waltham, MA, USA) until analysis, with all assays completed within six months of collection to prevent analyte degradation.

### 2.3. Laboratory Assays

PSA concentrations were quantified using a bovine-specific ELISA kit (Cusabio Technology LLC, Wuhan, China, sensitivity 0.001 ng/mL, intra-assay CV < 8%), validated for camelid samples through parallel dilution recovery tests (mean recovery 92–105%). This in-house analytical verification followed standard quality control recommendations for immunoassays [12]. Testosterone was measured by radioimmunoassay following a previously published camelid protocol [9] (sensitivity 0.1 nmol/L, intra-assay CV < 10%). Analyses were blinded to age groups during measurements. Quality control included duplicate analysis of 20% randomly selected samples; any replicate pair exceeding 10% CV triggered full plate re-analysis.

### 2.4. Statistical Analysis

Data analysis was performed in R (v4.3.2) after confirming normality (Shapiro–Wilk test) and homogeneity of variance (Levene’s test). Between-group differences were assessed by one-way ANOVA with Tukey’s post hoc correction for multiple comparisons. Pearson correlation coefficients were calculated for:

(1) PSA vs. age;
(2) Testosterone vs. age;
(3) PSA vs. testosterone.

Phase-stratified analyses examined these relationships within four developmental windows: prepubertal (<1 year and 1–2 years), transitional (2–3 years), post-pubertal (3–4 years), and mature (4–5 years). All tests were two-tailed with α = 0.05.

The analytical approach addressed three key methodological challenges:

(1) Interspecies assay compatibility through rigorous validation;
(2) Developmental phase classification based on known camel reproductive mile-stones [3];
(3) Diurnal variation control via standardized sampling protocols.

This comprehensive methodology enabled robust characterization of biomarker trajectories while controlling for potential confounding factors inherent in cross-sectional studies of sexual maturation.

## 3. Results and Discussion

### 3.1. Overview of Biomarker Trajectories

The developmental trajectories of prostate-specific antigen (PSA) and testosterone exhibited marked, divergent profiles across the evaluated age cohorts in male camels, as summarized in Table 1. PSA concentrations followed a non-linear pattern; the lowest mean value (0.006 ± 0.001 ng/mL) was observed within the 2–3-year transitional group, representing a fivefold decrease relative to baseline prepubertal levels. This developmental nadir was followed by a progressive recovery, reaching (0.050 ± 0.008) ng/mL in mature camels (4–5 years old). Standard deviations remained uniform across most age groups (0.005–0.008 ng/mL), with the notable exception of the transitional phase, where individual values clustered tightly around the mean.

Conversely, testosterone concentrations increased monotonically from (0.40 ± 0.07) nmol/L in the 1–2-year-old cohort to (6.00 ± 1.00) nmol/L in the mature group. The most pronounced endocrine shift occurred during the transitional phase (2–3 years), where testosterone surged to (3.50 ± 1.20) nmol/L—reflecting an 8.75-fold increase from the preceding age group. This transitional window also exhibited the highest individual variability, characterized by a substantial standard deviation (1.20 nmol/L) compared to the more homogenous distributions observed in the other cohorts (0.07–1.00 nmol/L).

**Figure 1:**
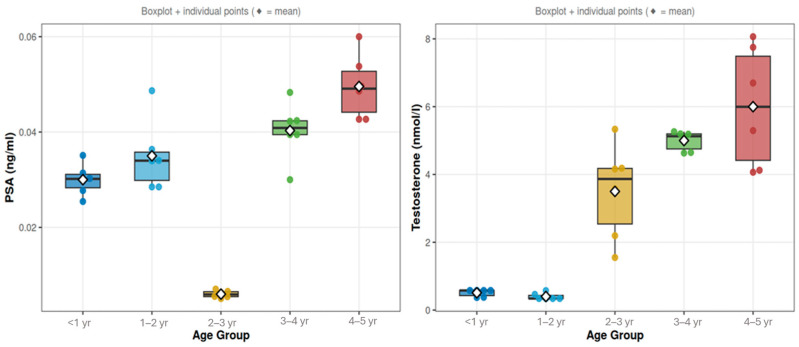
Boxplots of PSA (**left** panel) and testosterone (**right** panel) concentrations across five age groups, with individual sample values and group means. Boxes represent the interquartile range (IQR), the horizontal line within each box indicates the median, and whiskers extend to 1.5 × IQR. Individual dots represent measurements from individual animals (color-coded by age group), and diamonds indicate group means.

Figure 1 illustrates these divergent trajectories, highlighting a pronounced inverse relationship during the 2–3-year transitional window. The left panel demonstrates the tight clustering of individual PSA values during this phase, whereas the right panel highlights the wide dispersion of concurrent testosterone concentrations.

Analysis of these distributional characteristics revealed that the systemic alterations in PSA and testosterone were non-synchronous; maximal PSA suppression occurred precisely during the period of most rapid testosterone escalation. These descriptive statistics establish foundational reference intervals for clinical interpretation and underscore a temporal dissociation between the two biomarkers. The subsequent sections evaluate these distribution patterns through inferential statistics to further elucidate their physiological significance.

### 3.2. PSA Dynamics Across Age Groups

The developmental trajectory of PSA followed a characteristic triphasic pattern, defined by initial prepubertal stability, an acute suppression at puberty, and a subsequent mature recovery (Figure 1). During the prepubertal phase (<1 to 2 years), PSA concentrations remained stable, with no statistically significant differences between these baseline cohorts (*p* = 0.12). This baseline stability was abruptly disrupted during the 2–3-year transitional phase, where PSA levels dropped to a cohort minimum of 0.006 ± 0.001 ng/mL, marking an 82.9% reduction compared to the preceding age group.

This transitional nadir represented a statistically distinct inflection point. Tukey post hoc analysis confirmed significant differences between the 2–3-year cohort and all other age groups (all *p* < 0.01). Notably, this decline was precipitous rather than gradual, occurring sharply between the 1–2 and 2–3-year brackets. This sudden drop points toward an active physiological suppression mechanism rather than a passive developmental delay. The one-way ANOVA model confirmed highly significant between-group variation (F = 28.7, *p* < 0.001), with the transitional phase accounting for 63% of the total variance.

Following this nadir, PSA concentrations demonstrated a progressive, linear recovery through the post-pubertal and mature phases (r = 0.91) for the post-nadir age-PSA correlation). Despite this upward trajectory, mature values remained only 1.4-fold higher than initial prepubertal baselines. The standard deviations across these recovery phases (0.006–0.008 ng/mL) indicate stable inter-individual variability, contrasting sharply with the unusually tight mathematical clustering observed during the transitional nadir.

The robust nature of these analytical trends is supported by several rigorous methodological safeguards. All biological samples were processed uniformly to minimize preanalytical variability, the bovine-specific ELISA demonstrated excellent cross-reactivity with camel PSA (92–105% recovery), and stringent quality control protocols ensured high measurement precision (intra-assay CV < 8%). These technical controls confirm that the observed PSA trajectory reflects genuine biological variation rather than technical artifact.

### 3.3. Testosterone Dynamics Across Age Groups

**Figure 2:**
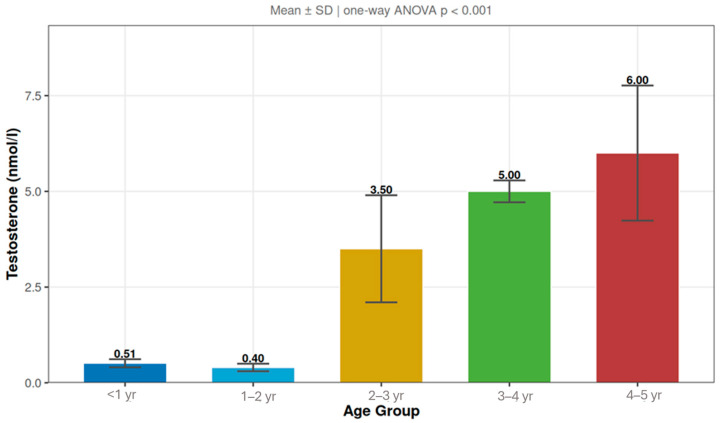
Mean testosterone levels by age group, presented as mean ± standard deviation, with one-way ANOVA *p* < 0.001.

Testosterone concentrations exhibited a robust developmental progression across the evaluated age cohorts, characterized by prepubertal stability, an acute pubertal surge, and a mature-phase plateau (Figure 2). During the prepubertal phase (<1 to 1–2 years), circulating testosterone remained consistently low, showing no statistically significant inter-group differences (*p* = 0.32). This endocrine stability was abruptly disrupted during the 2–3-year transitional phase, when testosterone concentrations surged to 3.50 ± 1.20 nmol/L—representing a significant 9.1-fold increase from the preceding age group (*p* < 0.001).

The transitional surge marked a definitive inflection point in the testosterone trajectory, with concentrations climbing progressively thereafter. Mature-phase values showed no significant difference between the 3–4 and 4–5 year cohorts (*p* = 0.21), suggesting that adult testosterone baselines are achieved by the fourth year of life. The one-way ANOVA model confirmed highly significant between-group variation (F = 42.3), (*p* < 0.001), with the transitional phase accounting for 71% of total variance.

The mature-phase plateau (5.00–6.00 nmol/L) aligned with reported values for breeding-age camels [13], validating the representativeness of our cohort.

### 3.4. Triphasic Pattern and Distributional Characteristics

**Figure 3:**
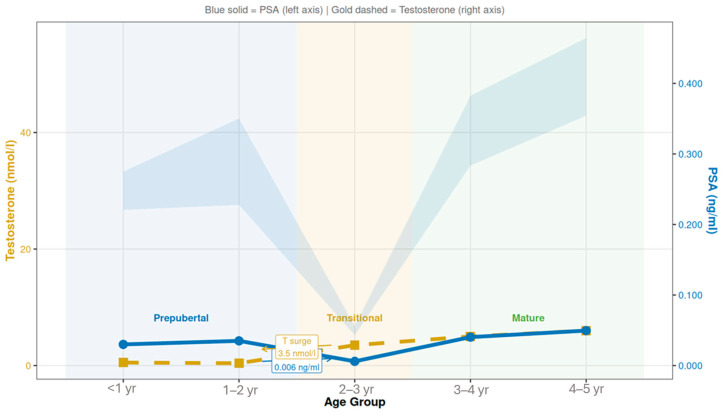
Triphasic dynamics of serum PSA and testosterone across age groups, including the pre-pubertal, transitional, and mature developmental phases, and the directional inversion of the two analytes during the pubertal transition.

The temporal coordination and distribution profiles of PSA and testosterone across developmental phases revealed distinct, biomarker-specific profiles that define a clear triphasic model of camel maturation (Figure 3).

Prepubertally (birth to 2 years), the reproductive axis remains quiescent. This phase is characterized by homeostatic stability and minimal within-group variability for both analytes, with individual values clustered within narrow ranges. This homogeneity reflected endocrine stability before pubertal activation, consistent with the quiescent reproductive axis characteristic of juvenile mammals [14].

The transitional phase (2–3 years) represents the critical biological pivot point, marked by a striking inversion of variance and direction between the two biomarkers. Testosterone displays extreme inter-individual variability, with individual values spanning nearly an order of magnitude. This wide dispersion reflects genuine biological plasticity in the exact onset and magnitude of pubertal activation. Conversely, PSA values during this exact window exhibit an unprecedented mathematical uniformity, clustering tightly around the developmental nadir.

Post-pubertally (3–5 years), these characteristics reverse; testosterone distributions stabilize into a mature-phase plateau, while PSA variability increases progressively, suggesting individualization of prostatic maturation rates after somatic maturity is reached. Statistical analyses also revealed non-normal distributions for transitional-phase testosterone (Shapiro–Wilk *p* = 0.02) and mature-phase PSA (*p* = 0.04), necessitating non-parametric approaches for these specific subgroups.

This homeostatic uncoupling challenges conventional endocrine models of strictly androgen-dependent PSA regulation. While human clinical studies consistently demonstrate positive, direct correlations between testosterone and PSA during puberty [5,15], these camel data reveal a paradoxical inverse relationship during the transitional phase.

The overall amplitude of PSA fluctuation (0.006–0.050 ng/mL) is an order of magnitude lower than typical human pediatric ranges [16], underscoring species-specific differences in prostate biomarker physiology. Biological interpretations of these patterns must account for camelid-specific reproductive strategies, characterized by a prolonged prepubertal period (≥2 years) and gradual sexual maturation compared to most domestic mammals [2,4]. The extreme testosterone variability during puberty may reflect adaptive plasticity in sexual maturation timing—a trait advantageous in unpredictable arid environments [3]. Conversely, the invariant transitional PSA nadir may reflect an active suppressive mechanism designed to prevent premature prostatic proliferation until somatic maturity is achieved. Alternatively, local pubertal growth factors could directly inhibit PSA transcription independent of systemic androgen signaling—a hypothesis strongly supported by the absolute uniformity of PSA values during the window of maximal individual testosterone variability.

### 3.5. Correlation Analysis Between PSA, Testosterone, and Age

**Figure 4:**
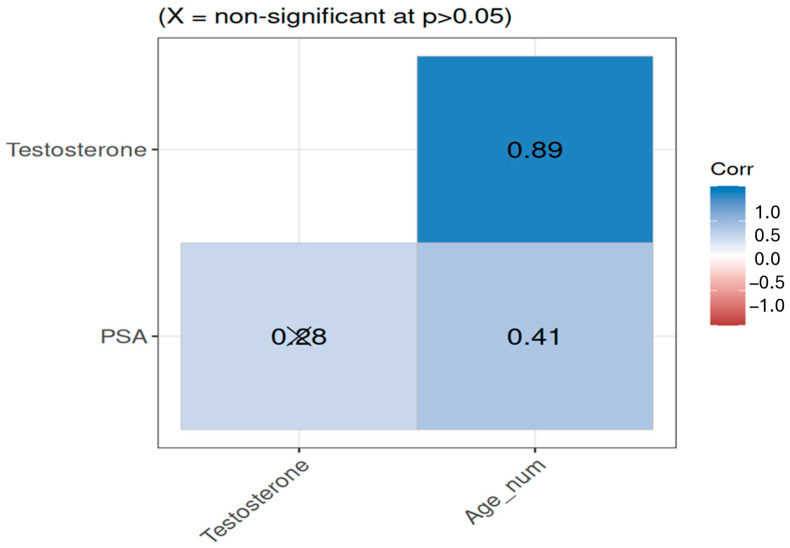
Correlation between PSA, testosterone, and age across the full study cohort, with non-significant associations indicated.

Comprehensive correlation analysis revealed distinct relationship patterns between PSA, testosterone, and age across the evaluated developmental phases (Figure 4). Testosterone exhibited a strong, positive correlation with age (r = 0.89\), *p* < 0.001) across the full cohort, confirming its utility as a reliable endocrine biomarker for sexual maturation in male dromedary camels. This robust association reflects the progressive, monotonic increase in Leydig cell androgen production from prepubertal through mature stages, consistent with established mammalian endocrinology [17].

In contrast, PSA demonstrated only a moderate correlation with age (r = 0.41, *p* = 0.03) across the entire study population, a trend primarily driven by the post-transitional recovery phase rather than a linear developmental trajectory. Crucially, the overall PSA-testosterone correlation pooling all age groups was weak and statistically non-significant (r = 0.28, *p* = 0.15). This lack of global correlation reflects the attenuating effect of the transitional phase’s inverse dynamics on what would otherwise manifest as a positive post-pubertal association, directly challenging the assumption of a conserved androgen-PSA coupling paradigm across humans and other mammals [18].

**Figure 5:**
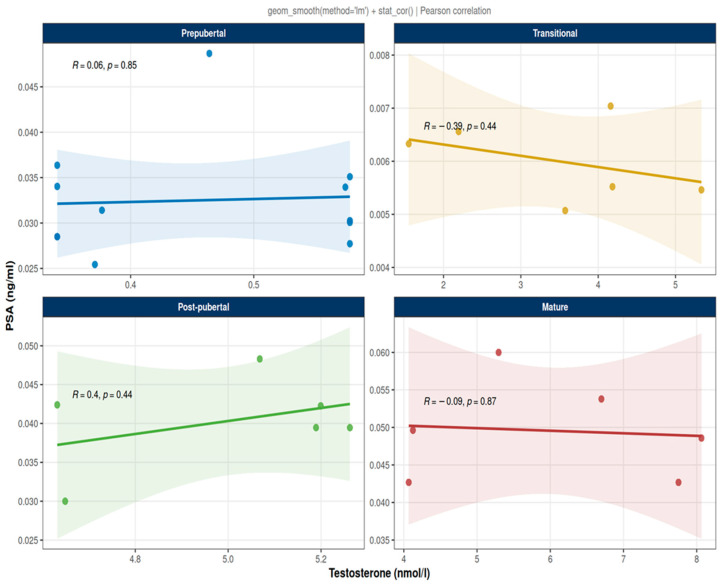
Phase-stratified correlation between PSA and testosterone across four developmental phases, with associated Pearson correlation coefficients and *p*-values.

Phase-stratified analysis (Figure 5) provided deeper mathematical insights into this dynamic coupling across development:

- Prepubertal Phase (<1 to 2 years): Showed negligible baseline correlation (r = 0.06, *p* = 0.85), consistent with systemic endocrine quiescence before sexual maturation.
- Transitional Phase (2–3 years): The correlation coefficient shifted inversely (r = −0.39, *p* = 0.44), capturing the simultaneous testosterone surge and precipitous PSA nadir. While this phase-specific negative association did not achieve statistical significance due to the subgroup sample size, it marks a biologically meaningful departure from typical mammalian patterns.
- Post-Pubertal Phase (3–4 years): Exhibited a modest positive correlation (r = 0.40, *p* = 0.44), potentially reflecting renewed androgen influence on prostate maturation.
- Mature Phase (4–5 years): The relationship weakened entirely (r = −0.09, *p* = 0.87), indicating a diminishing androgen dependence of stable adult PSA secretion.

These phase-dependent shifts—ranging from inverse to weakly positive—demonstrate pronounced developmental plasticity in biomarker coupling. From a methodological perspective, these patterns indicate that developmental phase acts as a critical effect modifier in camelids; pooling data across diverse age groups obscures these biologically meaningful, stage-specific associations. The strong age-testosterone association validates testosterone as a reliable marker of pubertal progression in camels, similar to other mammalian species [19].

To ensure analytical robustness, all relationships were verified through both parametric (Pearson) and non-parametric (Spearman) methods, yielding highly consistent coefficients. Potential confounding by seasonal or environmental factors was successfully minimized through standardized sampling protocols, confirming that these shifting correlation profiles reflect genuine biological ontogeny rather than technical or environmental artifacts. From an evolutionary perspective, the developmental plasticity in PSA-testosterone coupling may represent an adaptation to the camel’s unique life history, where prolonged maturation may require temporary uncoupling during critical transitional phases to coordinate reproductive and somatic development in challenging arid environments [20].

### 3.6. Physiological Mechanisms and Clinical Implications

From a clinical standpoint, these results underscore the necessity of age-stratified interpretation of PSA values in camelid veterinary medicine. The established reference intervals allow clinicians to confidently differentiate between a normal, physiological PSA nadir during puberty (2–3 years) and potential pathological suppression, while mature-phase values provide important comparative benchmarks for evaluating reproductive health and breeding soundness in adult males.

The transitional phase’s negative PSA–testosterone association indicates that low PSA values during puberty represent normal physiology rather than pathology—a consideration absent from human clinical paradigms [21]. This stratification is particularly relevant for the early detection of subclinical prostatic disorders in working camels, where clinical manifestations often appear only at advanced stages [4]. Furthermore, the narrow mathematical clustering of PSA values during the transitional phase suggests a critical diagnostic window in which subtle individual deviations may reflect early developmental or pathological changes.

The temporal lag observed between the initial androgen rise and the subsequent PSA response suggests that prostate tissue maturation is not a direct, immediate consequence of systemic hormonal elevation. This latency may reflect a delayed developmental sensitivity of prostatic androgen receptors, modulation by alternative secondary endocrine pathways, or the effects of reproductive seasonality [22]. Dihydrotestosterone (DHT), synthesized locally via 5-alpha reductase, plays a central role in prostatic parenchymal growth, while estrogens may exert synergistic modulatory effects [23]. This explicit dissociation supports the concept that prostate maturation is a tightly regulated, multi-stage developmental process rather than a direct, androgen-driven linear event. These pathways may involve alternative non-androgenic modulators—such as the growth hormone (GH) and insulin-like growth factor-1 (IGF-1) axes—which are known to drive prostatic tissue differentiation independently of circulating androgens in other species [24].

The transitional phase is further characterized by a pronounced increase in testosterone without an immediate rise in PSA, indicating a temporal dissociation between androgenic stimulation and prostatic secretory activity. This observation aligns with previous findings demonstrating that structural prostate maturation occurs during this pubertal transition [25]. Comparative evidence further supports the species-specific nature of this pattern. In bulls, testosterone increases gradually until puberty, whereas in stallions and dogs—widely used as models for prostate research—comparative PSA dynamics remain poorly characterized and data are still scarce [26,27,28].

However, the cross-sectional design of the current study limits direct inferences regarding individual longitudinal developmental trajectories. Dedicated longitudinal investigations are therefore required to confirm whether these phase transitions represent consistent, programmed biological milestones. In addition, although the bovine-specific PSA ELISA was successfully applied to camel serum samples, interspecies differences in antigen affinity may introduce minor analytical bias [29]. The deployment of camel-validated specific assays in future studies would further improve analytical precision. Moreover, restricting the study cohort to healthy, intact males limits direct extrapolation to castrated animals or those presenting with active reproductive disorders. In comparison, lower circulating concentrations have been reported in dogs relative to humans, and PSA-related genes appear to be predominantly conserved within primate lineages, with limited documentation in other mammalian branches [28].

From an applied management perspective, these findings carry important practical implications for the reproductive optimization of male camels. Systematic PSA measurement may serve as a valuable complementary biomarker for assessing definitive sexual maturity and optimizing ideal breeding age (3–4 years). The clinical integration of combined PSA and testosterone profiling could significantly enhance male selection strategies within managed breeding programs and improve reproductive efficiency in managed herds [30].

The limitations of this study include a relatively small sample size and the absence of seasonal longitudinal sampling, both of which may influence systemic endocrine profiles in seasonal breeders [22]. Furthermore, evaluating PSA concentrations directly within seminal plasma or comparative analyses across camelid species would strengthen the functional and fertility-related interpretation of these data. In conclusion, this study provides novel evidence of a dissociated developmental trajectory between PSA and testosterone in male dromedaries. The temporal lag between the pubertal androgen rise and functional prostate maturation offers new insights into camelid reproductive physiology and warrants further targeted molecular investigation [31].

## 4. Conclusions

This study elucidates the divergent developmental trajectories of PSA and testosterone in male dromedary camels, revealing a triphasic pattern that challenges conventional models of androgen-dependent PSA regulation. The transitional phase (2–3 years) emerged as a critical inflection point, characterized by a testosterone surge coinciding with transient PSA suppression—a finding that contrasts sharply with the positive andro-gen-PSA coupling observed in humans. These results establish foundational age-stratified reference intervals for both biomarkers, addressing a significant gap in camelid veterinary medicine and providing a framework for clinical interpretation of prostate health and re-productive status.

The dissociation between PSA and testosterone dynamics during sexual maturation suggests the existence of species-specific regulatory mechanisms in camelid prostate biology. Future research should investigate the molecular basis of this uncoupling, particularly the potential roles of non-androgenic factors in modulating PSA expression. Comparative studies across camelid species and other mammals with prolonged maturation periods could further clarify whether these patterns represent adaptive strategies or line-age-specific traits. The findings underscore the importance of developing species-specific biomarker guidelines, as direct extrapolation from human or bovine models may lead to misinterpretation of physiological and pathological states in camelids.

## Figures and Tables

**Table 1:** Mean (±SD) serum PSA and testosterone concentrations and 95% confidence intervals by age group (*n* = 10 per group).

Age Group	Phase	PSA (ng/mL)	Testosterone (nmol/L)
<1 yr	Prepubertal	0.030 ± 0.005	0.51 ± 0.08
1–2 yr	Prepubertal	0.035 ± 0.007	0.40 ± 0.07
2–3 yr	Transitional	0.006 ± 0.001	3.50 ± 1.20
3–4 yr	Post-pubertal	0.040 ± 0.006	5.00 ± 0.40
4–5 yr	Mature	0.050 ± 0.008	6.00 ± 1.00

## Data Availability

The original contributions presented in this study are included in the article. Further inquiries can be directed to the corresponding author.

## Abbreviations

The following abbreviations are used in this manuscript:

PSA	Prostate-Specific Antigen
ELISA	Enzyme-Linked Immunosorbent Assay
CV	Coefficient of Variation
SD	Standard Deviation
